# Annotations

**Published:** 1895-07-27

**Authors:** 


### Annotations.

### Eucalyptus Treatment at Leicester.

Some little time ago it was contended by Mr. Curgenven and others that eucalyptus oil by itself constituted a complete and successful treatment for scarlet fever, and that it also acted as a germicide which purified the skin and other tissues, and rendered unnecessary at least three weeks of the usual isolation period of scarlatina cases. If this contention had been justified by general experience, an important practical advance would have been made in the art of dealing with scarlet fever. Contentions of this order can only be proved by experiments, and by experiments conducted by a great number of persons, under a great variety of circumstances, and extended over a very wide area. Dr. Joseph Priestly, M.O.H., Leicester, has conducted one such series of experiments, and he publishes the results in his last Annual Report of the Health of the Borough. In all, Dr. Priestly treated 120 cases by the eucalyptus method, and 161 by the ordinary method. It is instructive, but not in all respects conclusive, to mark the results. In the first place, the "fever symptoms" were got rid of considerably earlier under "ordinary" than under "eucalyptus" treatment—a decidedly unexpected result. As over against this must be set the fact that the death-rate was lower under eucalyptus than under ordinary treatment—the former showing a mortality of only 1·6 per hundred, whilst the latter gave a mortality of 4·3. The percentage of sequelæ was smaller, but only a little smaller, under the eucalyptus than under the ordinary treatment, the cases suffering from serious sequelæ after ordinary treatment being 83·0 per cent., whilst those under eucalyptus numbered only 79·3. It may here be noted that the percentage of cases showing serious sequelæ seems to have been decidedly high under both treatments. On the subject of complete disinfection of the bodies of those suffering from scarlet fever within the short period of ten days or a fortnight, Dr. Priestly does not feel justified in offering a decided opinion. The only conclusion of a practical kind which we can deduce seems to be this, that many more experiments, on a much larger scale, must be made before any one of the many important problems connected with the question can be authoritatively solved.

### The Poplar Hospital in Danger.

The Poplar Hospital for Accidents is decidedly one of the most popular institutions in East London. But popular as it is, it cannot escape the ever-increasing pressure of the times. No sooner have the managers spent £25,000 in the erection of new and worthier buildings than they are threatened with a calamity which, if not arrested, will seriously damage the institution and its patients for all future time. At the rear of the hospital is a large open space, and to this "lung," together with the open space in front, is largely due the striking success of the hospital in its treatment of every variety of accident. The "lung" will soon be a lung no longer, unless the public will once more come to the rescue, and find something like £3,000 in addition to the amount paid for the new buildings. The "lung" is building ground, and it is "up for sale." If it be built upon, the hospital will be completely blocked in by small property with manifold chimneys, and in that case some of the wards, instead of being charged with fairly pure air, may be filled with smoke all the day long. Mr. Hills, the well-known president of the hospital, has promised £250 towards the £3,000 required, and the Hon. Sydney Holland, who has so often championed the institution in public, is well to the front in the hour of need. It is impossible for a newspaper which concerns itself with hospitals to speak too highly of the Poplar Hospital and its work. From the commencement of its life, more than forty years ago, until now, it has been an unmixed blessing to the strenuous daily toilers of East London and the docks. What the neighbourhood would have done without it we hardly dare to conjecture. With all possible earnestness we commend the hospital at its present crisis to the sympathies of intelligent philanthropists. We may take the opportunity of adding that an income of £6,000 a year is necessary for the full utilisation of the new buildings. There is no institution in London which more needs or better deserves a large addition to the list of its annual subscribers than the Poplar Hospital for Accidents.

### "Self-Help" and the "Heads of the Profession."

A few weeks ago a speaker at one of the medical societies urged upon his hearers the necessity of "self-help" in such matters as the raising and equalisation of fees, the putting down of unqualified practice, the prosecution of quacks, and the general purification and improvement of medicine as a profession. A subsequent speaker said that he entirely agreed with all that had been said, but that general practitioners "looked to the heads of the profession to do these things for them." Now, there are two sorts of persons who are variously looked upon as "heads" of the profession, namely, those who are at the head in point of success in practice, and those who may be considered to be at the head in point of notoriety. The first class simply cannot do anything towards the improvement of general practice, because they have not the time. Medicine is quite unlike every other calling in this particular, that it is absolutely and entirely personal; and the man who is largely successful has no time for any other work but the detailed and day to day duties of his personal practice. The successful "head," therefore, "cannot," we insist, devote either time or energy to the improvement of general practice. As for those "heads," who are "heads" by virtue of their pursuit of notoriety, they cannot do anything of real worth for the improvement of practice, because they do not come to the work with clean motives. Their object in life is notoriety: they "exploit" the general practitioner for their own ends. Who, then, is to be looked to for the improvement of general practice? The general practitioner himself; he, and no one else. It is the most familiar of all common-places that the man who helps himself in this world is best helped. Why should the medical practitioner suppose that he differs from all the rest of the world, and that it is his business to remain in a state of semi-childhood all his life, whilst he vainly looks to other people to do for him what the average grown-up man always does for himself? If general practitioners will set to work in a body to help themselves in those things in which they have hitherto expected the "heads of the profession" to help them, they will find real help. If they do not, they will certainly get no help at all from any quarter, and they will not deserve any.

